# Genome sequence of a *Trypanosoma evansi* isolate from a dog in Uruguay

**DOI:** 10.1128/mra.00568-26

**Published:** 2026-07-01

**Authors:** María Ximena Simón, Carlos Robello, Gonzalo Greif

**Affiliations:** 1 Laboratorio de Interacciones Hospedero-Patógeno–UBM, Institut Pasteur de Montevideo 123939, Montevideo, Uruguay; 2 Unidad Académica de Bioquímica, Facultad de Medicina, Universidad de la República 54795, Montevideo, Uruguay; Nanchang University, Nanchang, Jiangxi, China

**Keywords:** *Trypanosoma evansi*, surra disease

## Abstract

The parasite *Trypanosoma evansi* affects domestic and wild animals and is transmitted by blood-sucking insects. Among African trypanosomes, it has the widest global distribution, spanning Africa, Asia, and South America. Here, we report the genome assembly of the first isolate from Uruguay, obtained from a naturally infected dog.

## ANNOUNCEMENT

*Trypanosoma evansi* is the causative agent of surra disease in equines (1). The parasite also infects a wide range of hosts, including domestic (e.g., dogs) and wild animals (2). The presence of *T. evansi* in Uruguay was first reported by our group in a Cimarron dog from the north of the country in 2016 (3). Amplification and sequence analysis of the complete ITS1 region showed 100% identities to sequences of *T. evansi* isolated from the large, semiaquatic South American rodent capybara (accession no. KC988260) (4) and dog (accession no. KX926429) (5) reported in Argentina. The parasite was isolated from infected blood by inoculation into Balb/cJ mice and maintained cryopreserved in liquid nitrogen.

To obtain DNA for sequencing, parasites were expanded in the murine model under protocol CEUA 017-22. Mice were monitored daily by microscopic examination of blood smears, and parasites were collected when parasitemia reached its peak, as previously described (6). Parasites were then purified from mouse blood using a Percoll gradient (7). Approximately 1 × 10⁷ parasites were used for genomic DNA extraction using the Quick-DNA Miniprep Plus Kit (Zymo Research, USA), according to the manufacturer’s instructions. Genomic DNA was not sheared, and no size selection step was performed prior to library preparation. An Oxford Nanopore library was prepared using the Ligation gDNA Native Barcoding Kit v14 (SQK-NBD114-24, Oxford Nanopore). The final library (20 fM) was loaded onto the R10.4.1 flow cell (FLO-MIN114) and sequenced in a MinION MK1C for 24 h. Basecalling was performed using Guppy v7.1.4 with the dna_r10.4.1_e8.2_400bps_5khz_hac_mk1c.cfg model configuration (high-accuracy mode). Adapter and barcode trimming was performed during the Guppy demultiplexing step, and no additional read trimming was applied before assembly. The run produced 972.6 k passed reads (min *Q*-score >9, N50 = 4.96 kb, total yield = 4.92 Gb*)*, corresponding to an estimated genome coverage of approximately 115×.

Genome assembly was performed using Canu 2.2 (8) with these options: genomeSize = 40 m, correctedErrorRate = 0.105, minOverlapLength = 100, useGrid = false, maxThreads = 24 and minReadLength = 3,000*.*

The final assembly consists of 279 contigs (N50 = 682,065 bp, L50 = 15), with a GC content of 44.28% and a total length of 42.74 Mb. The largest contig is 4,705,273 bp. Assembly completeness was assessed using BUSCO v5.7.0 in genome mode with the euglenozoa_odb10 lineage data set, revealing 99.3% complete BUSCOs (93.1% single-copy and 6.2% duplicated), no fragmented BUSCOs, and only one missing BUSCO (0.7%). Annotation was performed using Companion v2.2.11 (9), using *Trypanosoma brucei* as a reference, resulting in 13,059 predicted genes, 88 tRNAs, 196 rRNAs, 66 snoRNAs, and 2 snRNAs. The *T. brucei* reference was selected because of its more comprehensive and well-curated annotation and its close phylogenetic relationship with *T. evansi.*

Unless otherwise specified, all software was run using default parameters.

## Data Availability

This whole-genome assembly project has been deposited in DDBJ/ENA/GenBank under accession no. JBXYWW000000000 (PRJNA1460257). The version described in this paper is the first version (JBXYWW010000000). Raw reads are available at NCBI GenBank under accession no. SRR38329285.
